# Thermodynamic coupling between cold and heat activations of TRPV2

**DOI:** 10.1038/s41598-026-54781-w

**Published:** 2026-06-10

**Authors:** Guangyu Wang

**Affiliations:** 1 Department of Physiology and Membrane Biology, University of California School of Medicine, Davis, CA USA; 2 Department of Drug Research and Development, Institute of Biophysical Medico-chemistry, Reno, NV USA

**Keywords:** Biophysics, Chemistry, Neuroscience, Physics

## Abstract

The homotetrameric thermosensitive transient receptor potential vanilloid 2 (TRPV2) channel is a biological macromolecule with unique high temperature threshold and sensitivity. However, the underlying thermodynamic basis has not been well understood. In this computational study, the 3D cryo-EM structures of rat TRPV2 in response to various chemical perturbations at different sites at low temperatures were quantified at the tertiary and quaternary levels using a highly sensitive thermoring model. The results indicated that a putative stable pre-open closed state without a lipid at the well-known active vanilloid site exhibited at least three weakest tertiary noncovalent bridges on the protein surface as primary thermal sensors with matched thresholds for initial heat activation. Any chemical perturbation away from these sensors activated the channel but with lower cold sensitivity. In contrast, when the sensors were simultaneously exposed to a mild detergent, together with hydrolysis of nearby charged residues at the membrane surface, the channel could be opened with the unique high cold sensitivity similarly to mirror the initial heat sensation. Further, disrupting intersubunit interactions near the heat sensors was required for full channel opening at both upper and lower gates. Therefore, the heat capacity mechanism, once cross-examined, could be applied to elucidate the unique thermoring basis for the sharp heat response of thermosensitive TRPV2 above body temperature.

**Supplementary Information:**

The online version contains supplementary material available at 10.1038/s41598-026-54781-w.

## Introduction

**Fig. 1:**
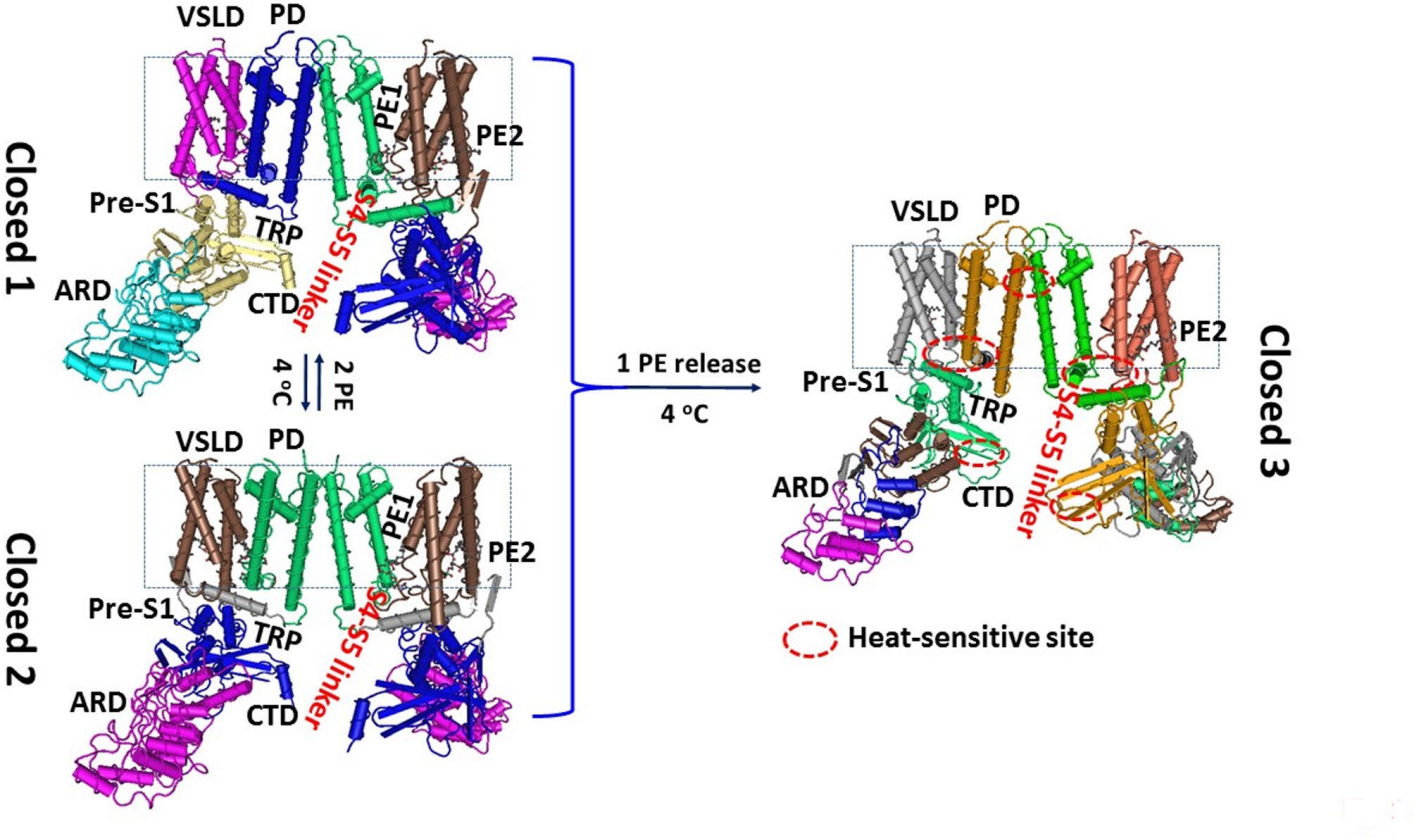
Structures of three closed states of rTRPV2 with PE-bound at low temperature. The cryo-EM structures of the rTRPV2 channel with PE in the VSLD or at the vanilloid site in the closed states 1, 2 and 3 at 4 °C (PDB: 8EKP, 8EKQ and 8EKR, respectively) were used for the model. For a convenient view, only two opposite subunits A and C are shown. Red circles indicate heat-sensitive phosphorylation or oxidation sites.

Heat-responsive homotetrameric thermosensitive transient receptor potential (TRP) vanilloid 1–4 (TRPV1-4) channels are biological macromolecules with multiple domains. Each monomer consists of the first four transmembrane helices (S1-S4) as the voltage sensor-like domain (VSLD) and the latter two (S5-S6) as the pore domain (PD), which includes two pore turrets and the pore helix (PH) between them. The S4-S5 linker is located at the active gating center, the TRP domain links S6 with the C-terminal domain (CTD), and the pre-S1 domain connects the VSLD to the ankyrin repeat domain (ARD). Along the centered pore, the selectivity filter has a GXGY motif (X = M, L; Y = D, E) and the lower gate is characterized by the LIA motif near the α-to-π transition of S6 (Fig. 1)^1^.

These membrane proteins have specific activation thresholds (T_th_) in response to heat stimuli. For example, 42 °C for rat TRPV1 (rTRPV1), 48–55 °C for rat TRPV2 (rTRPV2), 50 °C for mouse TRPV3 (mTRPV3) and 25–35 °C for human TRPV4 (hTRPV4). In addition, they exhibit a high temperature sensitivity Q_10_, which is the activity ratio of an ion channel assessed at temperatures 10 °C apart. For instance, Q_10_ > 20 for TRPV1 and TRPV3, 20 for TRPV4, and Q_10_ >100 for rTRPV2^2–20^. Although the structural basis for the initial heat responses of TRPV1 and TRPV3 has been well illustrated by temperature-dependent titration^21,22^, and oxidation of M528 in the S4-S5 linker and M607 at the selectivity filter or phosphorylation of Y335 in the Pre-S1-ARD linker, Y471 in the S2-S3 linker and Y525 in the S4-S5 linker has been shown to sensitize heat activation of rTRPV2 (Fig. 1)^15,17^, much less is known about the origin of the very high heat threshold and sensitivity of the rTRPV2 channel.

Recently, the weakest tertiary noncovalent interactions in cryogenic electron microscopy (cryo-EM) structures of TRPV1, TRPV3 and TRPV4 have been identified as primary thermal triggers with thresholds that match both theoretically and experimentally^19–31^. Furthermore, their unfolding at low or high temperatures, along with the broken swapping of Y565-R579’ or Y575-K589’ or Y602-R616’ π interactions near the lower gate, could ultimately activate the channels with mirrored cold and heat sensitivities, in accordance with the heat capacity mechanism^30–32^. Since the same mechanism applies to TRPV1-4 channels with some sequence homology, matched thresholds and thermosensitivities shared by both cold and heat activations from the same starters can be used to define primary thermal sensors in thermosensitive TRPV2, especially in the case that capturing their heat-evoked open states at higher temperatures remains challenging.

In this computational study, a highly sensitive thermoring model, recently developed^28–31,33–42^, was used to quantify the tertiary noncovalent interaction networks in the cryo-EM structures of rTRPV2 with and without phosphatidylethanolamine (PE) lipids at the vanilloid site and in the VSLD in various gating states upon chemical perturbations at low temperature^18,43,44^. After identifying at least three weakest tertiary noncovalent interactions on the protein surface as the primary thermo-sensors with matched thresholds for the initial heat activation and the differential scanning calorimetry (DSC) transition from a putative stable pre-open closed state without PE at the vanilloid site, various thermoring-based structural thermo-sensitivities were calculated and compared during cold activations in response to different chemical perturbations at low temperature. Although perturbations of phytocannabinoid Δ9-tetrahydrocannabiorcol (C16) or probenecid (PBC) away from the three putative superficial heat sensors triggered channel activations with a lower structural thermosensitivity, simultaneous exposure of sensors to a mild detergent, lauryl maltose neopentyl glycol (LMNG), favored hydrolysis of the nearby charged residues at the membrane-water interfaces for channel opening below the heat threshold but with a unique high structural thernosensitivity similarly to mirror the initial heat activation. Further, although disrupting intersubunit interactions near the heat-sensitive Y525 or M528 or M607 modification site was sufficient to initiate channel activation, swapping bridges near the external heat sensors must be broken for full channel opening at both upper (^606^GMGE^609^) and lower (^641^LI^642^) gates with high thermosensitivity. Therefore, this computational study suggested that the same heat capacity mechanism, once cross-examined, may also be involved, accounting for the very high heat sensitivity of rTRPV2.

## Methods

### Cryo-EM structures used

Three full-length cryo-EM 3D structures of the closed PE-bound rTRPV2 channels were examined as initial controls for cold or heat activation (PDB:8EKP for state 1, model resolution = 2.75 Å; 8EKQ for state 2, model resolution = 2.6 Å; 8EKR for state 3, model resolution = 3 Å, respectively)^44^. These channels were first purified in the decyl maltose neopentyl glycol (DMNG) detergent and then reconstituted in membrane scaffold protein 2N2 (MSP2N2) nanodiscs at 4 °C. In addition, the full-length cryo-EM 3D structures of C16- or PBC-activated TRPV2 in glyco-diosgenin (GDN) at 4 °C were used as controls to examine the heat capacity model (PDB: 7ZJD, model resolution = 2.9 Å; 7ZJE, model resolution = 3.2 Å; 7ZJG, model resolution = 3.0 Å)^18^. In contrast, the full-length cryo-EM 3D structure of the open PE-free rTRPV2 channel in the LMNG detergent at 4 °C was also studied to examine the symmetric cold and heat activations in the heat capacity model (PDB: 6BO4, model resolution = 4 Å)^43^.

### Detecting and filtering tertiary noncovalent interactions

The tertiary noncovalent interactions between two amino acid side chains or backbones, or combined along the PE-dependent minimal gating pathway of rTRPV2 from V254 to P726, were detected and filtered using UCSF Chimera with the same strict and consistent standards as previously confirmed^28–31,33–42^. Briefly, H-bonds were identified in the tertiary structure using UCSF Chimera, salt bridges were identified by scanning pairs of charged residues and lone pair/CH/cation/π-π interactions were identified by scanning aromatic residues such as Phe, Tyr or Trp and nearby residues. Specific cutoff distances and interaction angles for the different noncovalent interactions can be found in the online Supporting Information (Table S1, S2, S3, S4, S5, S6 and S7). It shoud be noted that momentary fluctuation-induced perturbations in tertiary noncovalent interactions during protein dynamics were not considered in this study.

### Mapping thermoring structures using the grid thermodynamic model

**Fig. 2:**
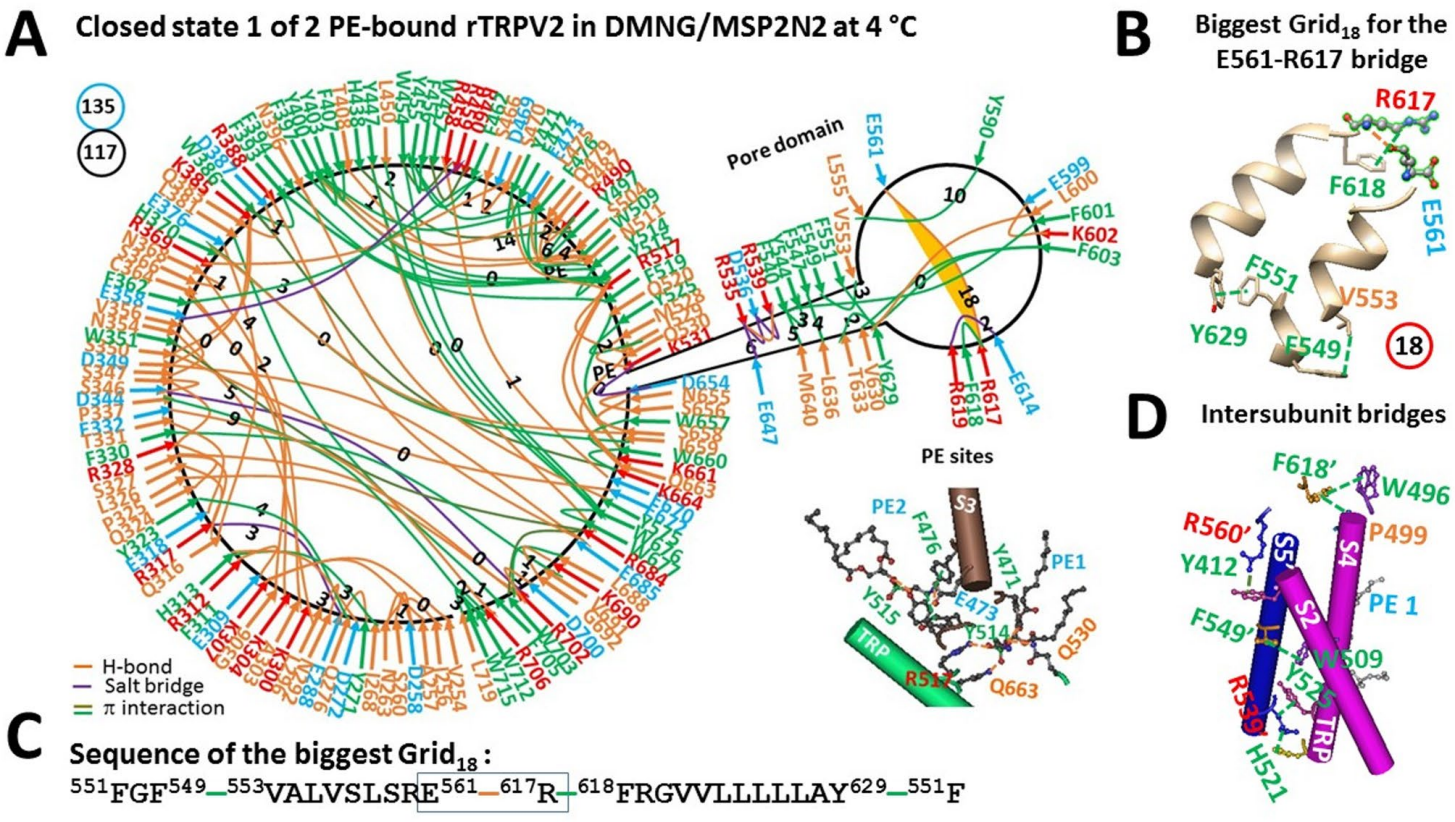
Thermoring structures of closed state 1 of 2 PE-bound rTRPV2 at low temperature. (**A**) The grid-like noncovalently interacting mesh network along the PE-dependent minimal gating pathway of a single subunit of the closed state 1 of rTRPV2 in DMNG/MSP2N2 nanodisc at pH 8 and 4°C (PDB: 8EKP). The same colored residues and arrows are used for convenient tracking. Salt bridges, π interactions, and H-bonds between paired amino acid side chains along the PE-dependent gating pathway from V254 to P726 are denoted in purple, green, and orange lines, respectively. The specific constrained grid sizes necessary to regulate the least-stable noncovalent interactions in the grids are indicated with black numbers. The identified least-stable E561-R617 H-bond in the biggest Grid_18_ is highlighted in orange. The total grid sizes and the total grid size-controlled noncovalent interactions along the PE-dependent minimal gating pathway are displayed in cyan and black circles, respectively. The inset shows the structure of the PE sites in the VSLD and at the vanilloid site. (**B**) The structure of the biggest Grid_18_ with an 18-residue size to regulate the weakest E561-R617 H-bond at the external interface of two pore turrets. (**C**) The sequence of the biggest Grid_18_ to control the weakest E561-R617 bridge highlighted in the blue box. (**D**) Swapping interactions at the VSLD/PD’/PD interfaces near the first PE site and upper and lower gates in closed state 1.

The study utilized the same protocol as previously described and validated to map the systematic fluidic grid-like noncovalent interaction mesh network as the thermoring structure[28–31,33–42]. First, all identified tertiary noncovalent interactions were classified into two groups: one in the pore domain (PD) and the other in the remaining domains such as ARD, pre-S1 domain, VSLD, TRP domain and the CTD. Second, along the defined PE-dependent minimal gating pathway of rTRPV2 from V254 to P726 (black line), all the involved amino acid residues were represented by arrowed nodes and all identified tertiary noncovalent interactions were represented as edges to link a pair of arrowed nodes. Thus, each eadg and nearby peptide segments with or without other eadgs could form several rings as topological grids but without repeating the same edge. As an example in Fig. 2A, the E599-K602 H-bond and the segment ^600^LF^601^ could generate a ring. Alternatively, this H-bond could form another ring with the nearby L600-Y629 H-bond and F603-Y629 π−π interaction (Fig. 2A). In this case, it was necessary to use Graph theory and the Floyd–Warshall algorithm to constrain these grids so that a unique grid could be obtained for each specific tertiary noncovalent interaction with the shortest round path length or the minimal number of free residues in it defined as the grid size^45^. As the melting temperature to unfold the least-stable tertiary noncovalent interaction in a grid is related to this unique grid size, the constained grid was also defined as a thermoring to control the least-stable interaction and denoted as Grid_s_. For example, in Fig. 2A, the least-stable E614-R619 π bridge was controled by the smallest Grid_2_ via a thermoring from E614 to R617, F618, R619 and back to E614 along the peptide segment ^615^QL^616^, the R617-F618 π bridge and the R619-E614 salt bridge while the R617-F618 was governed by the smallest Grid_0_ (Fig. 2A). In this manner, when all the tertiary noncovalent bridges were labeled by unshared grid sizes that corresponded to the minimum energy required to stabilize the interactions, the biggest thermoring could be identified to govern the weakest tertiary bridge along the PE-dependent minimal gating pathway from V254 to P726. Meanwhile, the total numbers of noncovalent interactions (*N*) and total grid sizes (*S*) along the PE-dependent minimal gating pathway of rTRPV2 from V254 to P726 were calculated and displayed in black and cyan circles, respectively, next to the mesh network map for the following calculations.

### Calculating the systematic thermal instability

The systematic thermal instability (T_i_) was calculated using the following equation as previously examined^28–31,33–42^:

1 $$T_{i} = S/N$$

### Calculating the melting temperature threshold for heat unfolding

The melting temperature threshold (T_m, th_) for the heat unfolding of the least-stable noncovalent interaction within a specific grid at the normal salt concentration of 150 mM NaCl was calculated using the following equation as previously examined^28–31,33–42^:

2 $$T_{{m,th}} ({}^{^\circ }C) = 34{\text{ }} + \left( {n - 2} \right) \times 10 + \left( {20{\text{ }}{-}{\text{ }}s} \right) \times 2$$

where, n represents the total number of basic H-bonds (~ 1 kcal/mol for each in a hydrophilic environment) that are energetically equivalent to the least-stable noncovalent interactions controlled by the given grid, and s is the size of the grid that controls the least-stable noncovalent interaction. Therefore, the heat capacity of the grid will increase along with a decrease in the grid size or an increase in the number of equivalent basic H-bonds.

### Evaluating the systematic temperature sensitivity of rTRPV2

A gating transition of the thermosensitive TRPV1, TRPV3 or TRPV4 channel is always accompanied by a series of local decyclizations and recyclizations along the lipid-dependent minimal gating pathway^28–31^. Accordingly, for enthalpy-driven activation of TRPV2 from a closed state within a 10 °C range due to the unfolding or refolding of the weakest bridges, if the chemical potential of a grid is theoretically defined as the maximal potential for equivalent residues in the grid to form the tightest β-hairpin with the smallest loop via noncovalent interactions^46^, the grid-based structural thermo-sensitivity (Ω_10_) of a single ion channel for cold inactivation could be defined and calculated using the following equations as examined previously^28–31,42^.

3 $$\Omega _{{10}} = \left[ {\left( {S_{c} {-}{\text{ }}S_{o} } \right)E/2} \right]^{{(Hc/Ho)}} = \left[ {\left( {S_{c} {-}{\text{ }}S_{o} } \right)E/2} \right]^{{[(ENc/(ENo)]}} = \left[ {\left( {S_{c} {-}{\text{ }}S_{o} } \right)E/2} \right]^{{(Nc/No)}}$$

where, along the same defined PE-dependent minimal gating pathway of one subunit from V254 to P726, N_c_ and N_o_ represent the total noncovalent interactions, H_c_ and H_o_ denote the total enthalpy included in them, and S_c_ and S_o_ indicate the total grid sizes in the closed and open states, respectively. The average energy intensity of a tertiary noncovalent interaction is denoted by E and is typically 1 kcal/mol^47^. Thus, the mean Ω_10_ factually reflects a thermo-evoked change in the total chemical potential of grids upon a thermo-evoked change in the total enthalpy included in the noncovalent interactions apparently from a closed state to an open state along the same defined PE-dependent minimal gating pathway of one subunit.

For a convenient comparison, the functional thermo-sensitivity (Q_10_) of a single ion channel for heat activation was calculated using the following equation:

4 $$Q_{{10}} = {\text{ }}\left( {X_{2} /X_{1} } \right)^{{10/(T2 - T1)}}$$

where, X_1_ and X_2_ are the relative channel activity obtained at temperatures T1 and T2 (measured in Kelvin), respectively.

## Results

### Dynamic PE lipid at the active vanilloid site in the resting closed state

The closed rTRPV2 channel exhibited three states when purified in the DMNG detergent and then reconsituted in MSP2N2 nanodiscs at 4 °C (Fig. 1)^44^. A PE lipid in these three states was anchored by F476 on S3 and Y514 and Y515 on S4 in the VSLD via H-bonds and π interactions (Fig. 2A). In addition, another PE lipid was present at the vanilloid site in states 1 and 2 but absent in state 3 (Fig. 1). It was stabilized by heat-sensitive Y471 on the S2-S3 linker, R517 and Q530 on the S4-S5 linker, Q663 on the TRP domain through several H-bonds and π interactions (Fig. 2A). Therefore, the vanilloid site lipid is dynamic even in the resting closed state at low temperature. Given that the heat-sensitive oxidation sites at M528 and M607 and phosphorylation sites at Y335, Y471 and Y525 are distributed across the entire protein (Fig. 1)^15,17^, it is necessary to quantify relevant tertiary and quaternary structures of rTRPV2 using the highly-sensitive thermoring model to reveal the thermodynamic basis for the high heat threshold and sensitivity.

### Identification of a putative stable pre-open closed state with matched thresholds for the initial heat activation

In the presence of the PE lipid at the active vanilloid site in state 1 (PDB: 8EKP), a total of 117 tertiary noncovalent interactions were generated mainly by side chains of amino acids along the PE-dependent minimal gating pathway from V254 to P726. When they formed a local grid-like mesh network, the total number of grid sizes was 135 (Fig. 2A; Table S1). Thus, the systematic thermal instability (T_i_) was calculated to be about 1.15 (Table 1). Meanwhile, the biggest Grid_18_ was found to control the weakest E561-R617 H-bond at the external interface of two pore turrets via a thermoring from E561 to R617, F618, Y629, F551, F549, V553 and back to E561 (Fig. 2A-C). When this least-stable bridge was energetically equivalent to 1.5 basic H-bond (1.5 kcal/mol), the melting threshold (T_m, th_) to unfold it was calculated as about 33°C, much lower than the initial threshold of 48–55°C for heat sensing (Table 1)^3,11–13,15–18,20^. Notably, H521 and Y525 on the S4-S5 linker from one subunit formed a CH-π interaction with R539’ on S5 from a neighbouring subunit (Fig. 2D). Given the first PE site above the S4-S5 linker and between the VSLD and the pore domain, these swapping bridges were related to not only the heat-sensitive phosphorylation sites at Y471 and Y525 but also the heat-sensitive oxidation site at M528 (Figs. 1 and 2A). Hence, they are essential for channel closure at the lower gate. Similarly, the swapping W496-F618’-P499 and Y412-R560’ π interactions were also present at the VSLD/PD’ interfaces for channel closure at the selectivity filter or the upper gate (Fig. 2D). In this case, it is reasonable to observe the swapping W509-F549’ bridges between the upper and lower gates (Fig. 2D).

**Table 1:** Comparison of the thermoring structures of *apo* rTRPV2 along the PE-dependent minimal gating pathway from V524 to P726. The comparative parameters are highlighted in bold.

PDB ID	8EKP	8EKQ	8EKR		
Construct	rTRPV2				
Lipid in the VSLD	bound				
Lipid at the active vanilloid site	bound		free		
Lipid environment	MSP2N2				
Sampling temperature, °C	4				
Gating state	Closed 1	Closed 2	Closed 3		
# of the biggest Grids	Grid_18_	Grid_14_	Grid_10_	Grid_7_	Grid_6_
grid size (s)	18	14	10	7	6
# of basic H-bonds (n) in stability equivalent to the weakest noncovalent bridge	1.5	2.0	1.4	1.3	0.56
Total non-covalent interactions (N)	117	115	107		
Total grid sizes (S), a.a	135	95	118		
Systemic thermal instability (Ti)	1.15	0.83	1.10		
Calculated T_m, th_ °C	**33**	**46**	**48**	**53**	**47.6**
Measured threshold T_th_, °C	**34.5**	**46**	**48–55**		
Refs for T_th_	^3,11–13,15–18,20^				

**Fig. 3:**
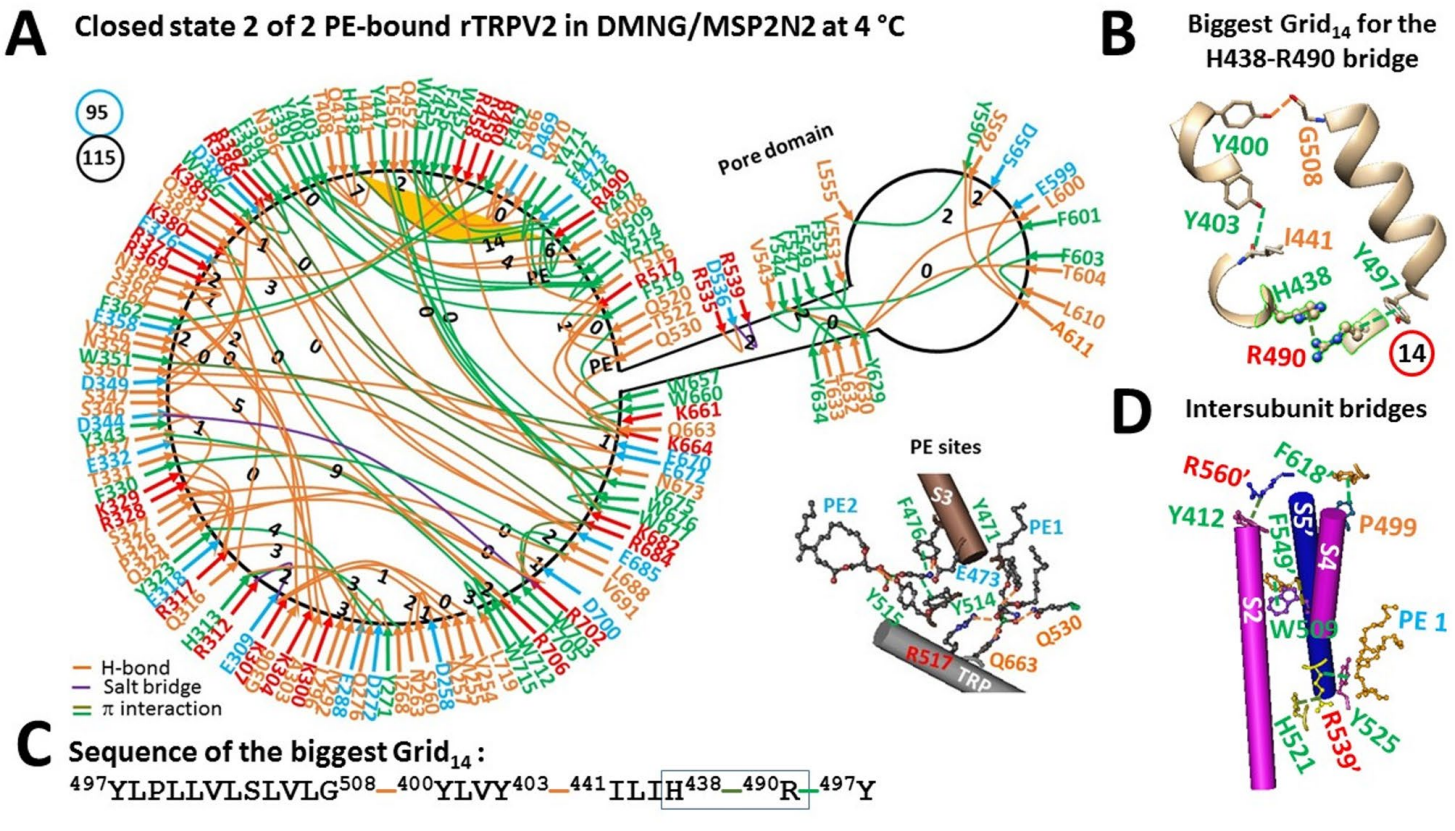
Thermoring structures of closed state 2 of 2 PE-bound rTRPV2 at low temperature. (**A**) The grid-like noncovalently interacting mesh network along the PE-dependent minimal gating pathway of a single subunit of the closed state 2 of rTRPV2 in DMNG/MSP2N2 nanodisc at pH 8 and 4°C (PDB: 8EKQ). The same colored residues and arrows are used for convenient tracking. Salt bridges, π interactions, and H-bonds between paired amino acid side chains along the PE-dependent gating pathway from V254 to P726 are denoted in purple, green, and orange lines, respectively. The specific constrained grid sizes necessary to regulate the least-stable noncovalent interactions in the grids are indicated with black numbers. The identified weakest R490-H438 cation-π bridge in the biggest Grid_14_ is emphasized in orange. The total grid sizes and the total grid size-controlled noncovalent interactions along the PE-dependent minimal gating pathway are displayed in cyan and black circles, respectively. The inset shows the structure of the PE sites in the VSLD and at the vanilloid site. (**B**) The structures of the biggest Grid_14_ with 14-residue sizes to regulate the weakest R490-H438 bridge at the external S1-S2 linker/S3-S4 linker interface. (**C**) The sequences of the biggest Grid_14_ to control the weakest R490-H438 bridge highlighted in the blue box. (**D**) Swapping interactions at the VSLD/PD’/PD interfaces near the first PE site and upper and lower gates in closed state 2.

In state 2 (PDB: 8EKQ), tertiary noncovalent interactions mainly formed by side chains of residues along the PE-dependent minimal gating pathway from V254 to P726 decreased to 115 along with the total number of grid sizes decreasing to 95 (Fig. 3A; Table S2). Thus, the T_i_ also decreased to about 0.83 (Table 1). Among these grids, the biggest Grid_14_ was present to control the weakest H438-R490 cation-π interaction at an external interface between S1-S2 and S3-S4 linkers via a thermoring from Y497, G508, Y400, Y403, I441, H438, R490, and back to Y497 (Fig. 3A-C). As this weakest bridge was energetically equivalent to 2.0 basic H-bonds (2.0 kcal/mol), the T_m, th_ was about 46°C, still less than the initial threshold of 48–55°C for the initial heat activation (Table 1)^3,11–13,15–18,20^. Meanwhile, along with the same swapping W509-F549’ bridges in the middle, the same swapping H521-R539’-Y525 and P499-F618’ and Y412-R560’ π interactions were located near the lower and upper gates and the first PE site, relating to the heat-sensitive phosphorylation sites at Y471 and Y525 and oxidation sites at M528 and M607 (Figs. 1 and 3A and D)^15,17^.

**Fig. 4:**
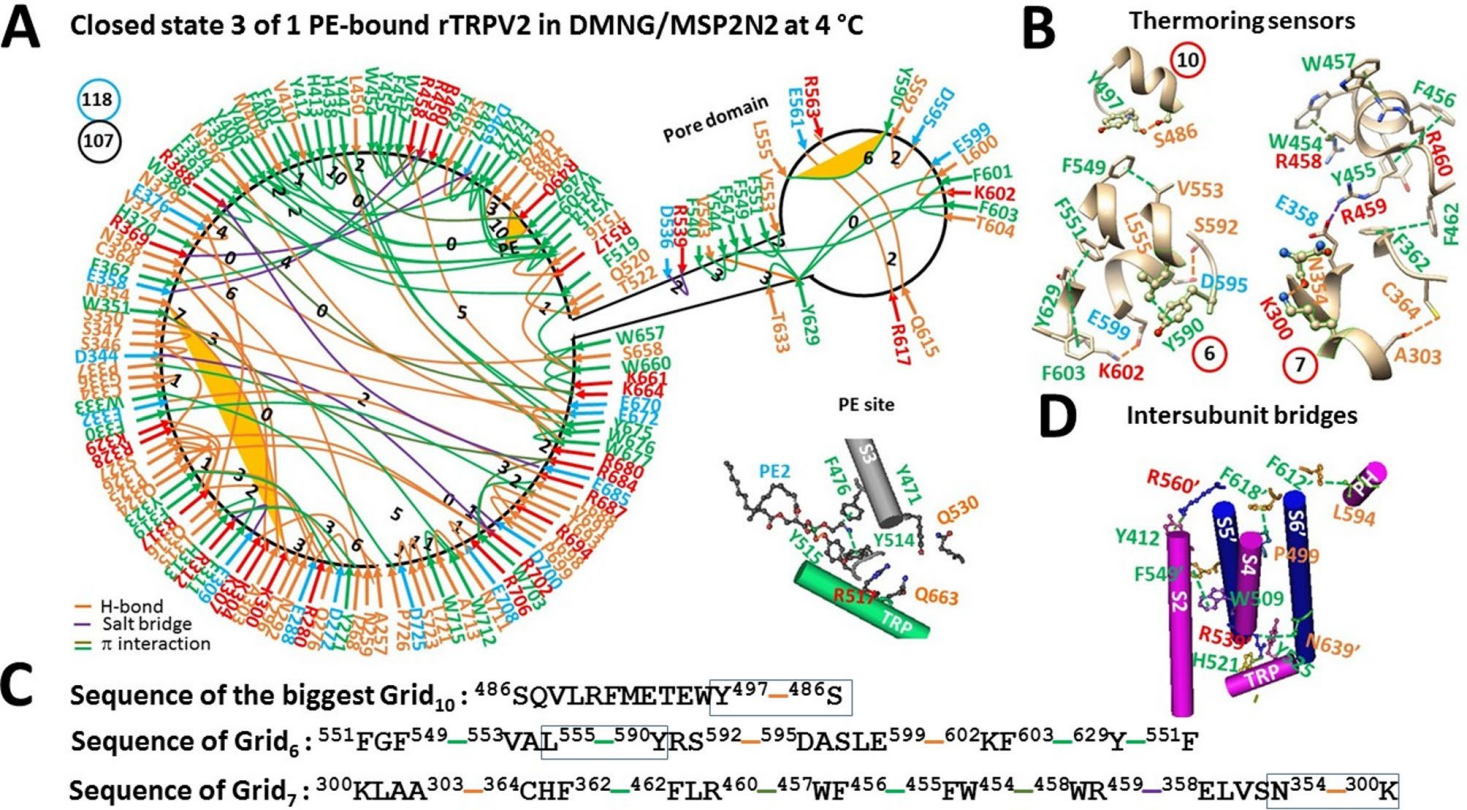
Thermoring structures of closed state 3 of 1 PE-bound rTRPV2 at low temperature. (**A**) The grid-like noncovalently interacting mesh network along the PE-dependent minimal gating pathway of a single subunit of the closed state 3 of rTRPV2 in DMNG/MSP2N2 at pH 8 and 4°C (PDB: 8EKR). The same colored residues and arrows are used for convenient tracking. Salt bridges, π interactions, and H-bonds between paired amino acid side chains along the PE-dependent minimal gating pathway from V254 to P726 are denoted in purple, green, and orange lines, respectively. The specific constrained grid sizes necessary to regulate the least-stable noncovalent interactions in the grids are indicated with black numbers. The identified weakest S486-Y497, K300-N354 and L555-Y590 bridges in the primary biggest Grid_10_, Grid_7_ and Grid_6_ are emphasized in orange. The total grid sizes and the total grid size-controlled noncovalent interactions along the PE-dependent minimal gating pathway are displayed in cyan and black circles, respectively. The inset shows the structure of the PE site in the VSLD and at the vanilloid site. (**B**) The structures of the primary biggest Grid_10_, Grid_7_ and Grid_6_ with 10, 7 and 6-residue sizes to regulate the weakest S486-Y497, K300-N354 and L555-Y590 bridges at various external and internal interfaces. (**C**) The sequences of the primary biggest Grid_10_, Grid_7_ and Grid_6_ to control the weakest S486-Y497, K300-N354 and L555-Y590 bridges highlighted in the blue boxes. (**D**) The swapping bridges at the VSLD/PD’/PD interfaces near the upper and lower in closed state 3.

In contrast, when the PE lipid was dynamically released from the active vanilloid site in state 3 (PDB, 8EKR), tertiary noncovalent interactions mainly formed by side chains of residues along the PE-dependent minimal gating pathway from V254 to P726 significantly decreased to 107 but the total number of grid sizes increased to 118 concurrently (Fig. 4A; Table S3). Thus, the T_i_ increased to 1.10 (Table 1), suggesting a stable closed state even without PE at the vanilloid site. Meanwhile, three biggest thermorings emerged. The primary biggest Grid_10_ appeared to govern the weakest S486-Y497 H-bond in the VSLD via a thermoring from S486 to Y497 and back to S486 (Fig. 4A-C). Because this H-bond was energetically equivalent to 1.4 basic H-bonds (1.4 kcal/mol), the calculated T_m, th_ was about 48 °C, falling within the measured threshold range of 48 °C to 55 °C for the initial heat activation (Table 1)^3,11–13,15–18,20^. The second biggest Grid_7_ was identified to control the weaker K300-N354 H-bond at the interface between the pre-S1 domain and the ARD (Fig. 4B). It encompassed a 7-residue size via a thermoring from K300 to A303, C364, F362, F462, R460, W457, F456, F455, W454, W458, R459, E358, N354, and back to K300 (Fig. 4B-C). With the weaker H-bond being energetically equivalent to 1.3 basic H-bonds (1.3 kcal/mol), the calculated T_m, th_ was about 53 °C (Table 1), close to the measured threshold range of 48 °C to 58 °C for heat activation^3,11–13,15–18,20^. In addition to the first two biggest Grid_10_ and Grid_7_ (Figs. 4A-C), the third biggest Grid_6_ was located in the pore domain to control the weakest L555-Y590 π interaction via a thermoring from L555 to V553, F549, F551, Y629, F603, K602, E599, D595, S592, Y590, and back to L555 (Fig. 4A-C). When 0.56 equivalent basic H-bonds sealed the weakest L555-Y590 π interaction, the calculated T_m, th_ was about 47.6 °C (Table 1), consistent with the measured threshold range of 48 °C to 55 °C as well for initial heat activation and DSC transition^3,11–13,15–18,20^. Therefore, at least three weakest bridges such as S486-Y497, K300-N354 and L555-Y590 in the biggest Grid_10_, Grid_7_ and Grid_6_ may serve as primary heat sensors of rTRPV2 for the initial heat activation with matched temperature thresholds.

Notably, despite the release of a PE lipid from the active vanilloid site, the channel remained closed. Hence, in addition to the swapping of Y412-R560’, P499-F618’, W509-F549’ and H521-R539’-Y525 bridges, it is reasonable to observe that the additional Y525-N639’ and L594-F612’ π bridges were formed near the lower and upper gates, relating to the heat-sensitive oxidation or phosphorylation site at Y525, M528 or M607 (Figs. 1 and 4D)^15,17^. On the other hand, when the total grid sizes in the first two closed states were averaged at 115, which was similar to the value in the third one (Table 1), the mean structural thermosensitivity (Ω_10_) was calculated as about -1.55 upon the release of PE from the vanilloid site. Thereafter, it is also reasonable that no significant change in heat capacity was observed before 47.7 °C^20^.

### External chemical perturbation away from the heat sensors induces weaker cold activation

Given that the identified heat sensors emerged near the ARD and in both the VSLD and the pore domain (Fig. 4A-B), it is particularly interesting to investigate if a chemical perturbation at the vanilloid site between the VSLD and the pore domain can induce cold activation to mirror the heat activation.

**Fig. 5:**
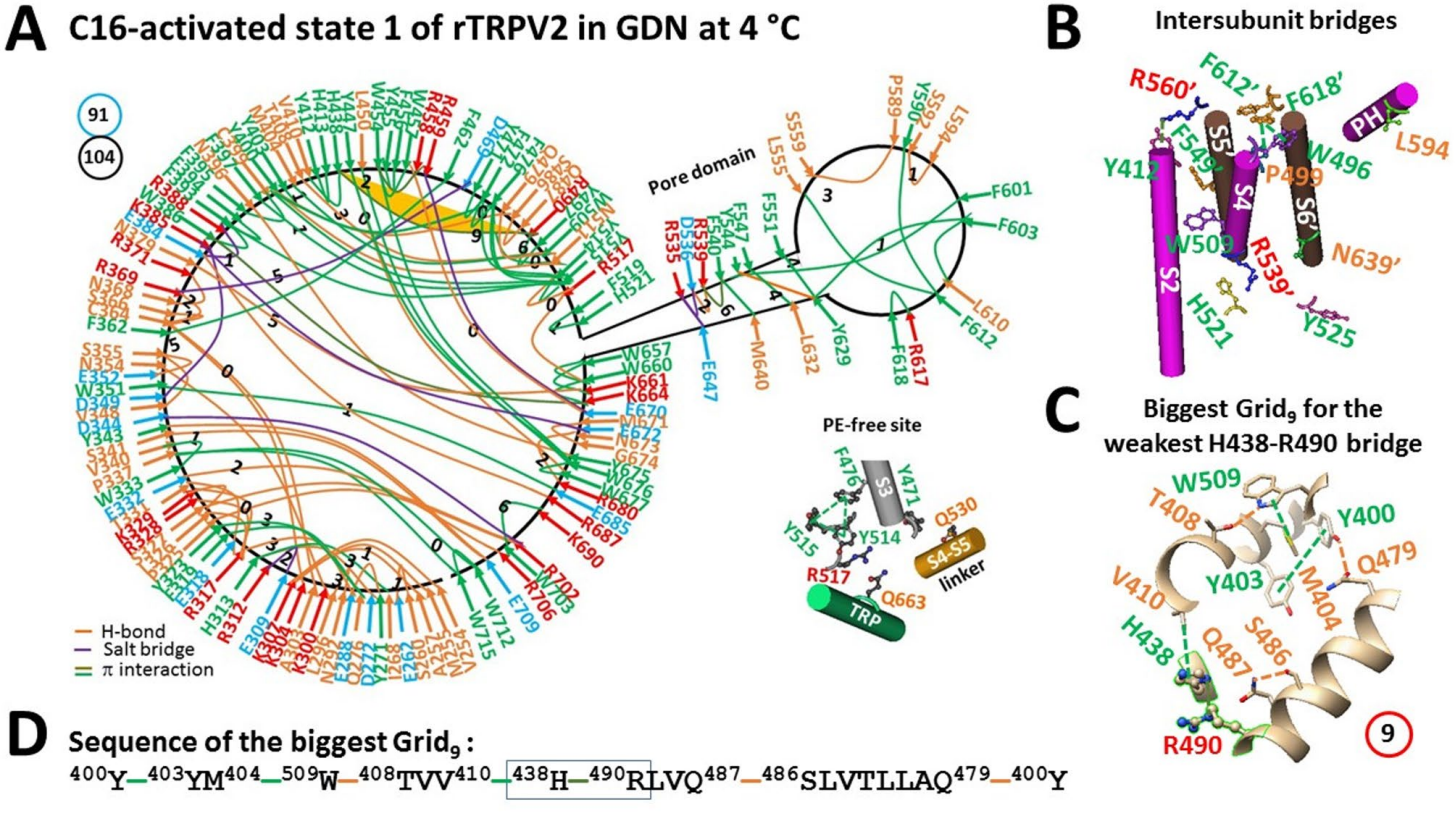
Thermoring structures of C16-activated state 1 of PE-free rTRPV2 at low temperature. (**A**) The grid-like noncovalently interacting mesh network along the PE-dependent minimal gating pathway of a single subunit of C16-activated state 1 of rTRPV2 in GDN at pH 8 and 4°C (PDB: 7ZJD). The same colored residues and arrows are used for convenient tracking. Salt bridges, π interactions, and H-bonds between paired amino acid side chains along the PE-dependent minimal gating pathway from V254 to P726 are denoted in purple, green, and orange lines, respectively. The specific constrained grid sizes necessary to regulate the least-stable noncovalent interactions in the grids are indicated with black numbers. The weakest H438-R490 cation-π bridge in the biggest Grid_9_ is emphasized in orange. The total grid sizes and the total grid size-controlled noncovalent interactions along the PE-dependent minimal gating pathway are displayed in cyan and black circles, respectively. The inset shows the structure of the PE-free site in the VSLD and at the vanilloid site. (**B**) Swapping interactions at the VSLD/PD’/PD interfaces near upper and lower gates along with the PE release in the C16-activated state 1. (**C**) The structure of the biggest Grid_9_ with a 9-residue size to regulate the weakest H438-R490 cation-π bridge at the external S1-S2 linker/S3-S4 linker interface. (**D**) The sequence of the biggest Grid_9_ to control the weakest H438-R490 cation-π bridge highlighted in the blue box.

With C16 targeting S526 on the S4-S5 linker, D536 and F540 on S5 and L636 on S6, two activated states were observed^18^. In the first activated state, following the release of PE from the VSLD (Fig. 5A), the swapping H521-R539’-Y525-N639’ and L594-F612’ bridges were also disrupted but the Y412-R560’ and P499-F618’-W496 bridges were not (Fig. 5B). In this case, when the weakest S486-Y497 bridge in the VSLD and the weakest L555-Y590 bridge in the pore domain were disrupted, the weakest H438-R490 cation-π bridge at the interface between S1-S2 and S3-S4 linkers was identified in the biggest Grid_9_ (Fig. 5A). It had a 9-residue size via a thermoring from Y400 to Y403, M404, W509, T408, V410, H438, R490, Q487, S486, Q479, and back to Y400 (Fig. 5C-D). When the least-stable salt bridge was energetically equivalent to 1.9 basic H-bond (1.9 kcal/mol), the calculated T_m, th_ was about 55 °C (Table 2). On the other hand, when the total tertiary noncovalent bridges and grid sizes decreased from 107 and 118 to 104 and 91, respectively (Figs. 4A and 5A; Tables S3 & S4), the mean structural thermosensitivity (Ω_10_) was calculated as about 14.6 (Table 2), much less than the initial heat sensitivity (Q_10_) of 132^12^. As a result, in the C16-activated state 1, the chemical perturbation away from the putative heat sensors at low temperature was insufficient to induce cold activation with matched thermosensitivity to mirror the initial heat activation.

**Table 2:** Comparison of chemically-induced thermoring structural changes of rTRPV2 along the PE-dependent minimal gating pathway from V524 to P726 at low temperature. The comparative parameters are highlighted in bold.

PDB ID	7ZJD	7ZJE	7ZJG	6BO4
Construct	rTRPV2			
Lipid in the VSLD	free			
Lipid at the active vanilloid site	free			
Lipid environment	GDN			LMNG
Sampling temperature, °C	4			
Gating state	Activated	Activated	Activated	Open
# of the biggest Grids	Grid_9_	Grid_21_	Grid_9’_	Grid_14’_
grid size (s)	9	21	9	14
# of basic H-bonds (n) in stability equivalent to the weakest noncovalent bridge	1.9	1.0	1.0	1.5
Total non-covalent interactions (N)	104	87	83	55
Total grid sizes (S), a.a	91	114	91	94
Systemic thermal instability (Ti)	0.88	1.31	1.10	1.71
Calculated T_m, th_ °C	**55**	**32**	**46**	**41**
Measured threshold T_th_, °C				
Calculated Ω_10_, mean at E = 1.0 kcal/mol	**14.6**	**2.35**	**28.6**	**126**
Measured Q_10_	**132**			
Refs for T_th_ and Q_10_	^12^	^12^	^12^	^12^

**Fig. 6:**
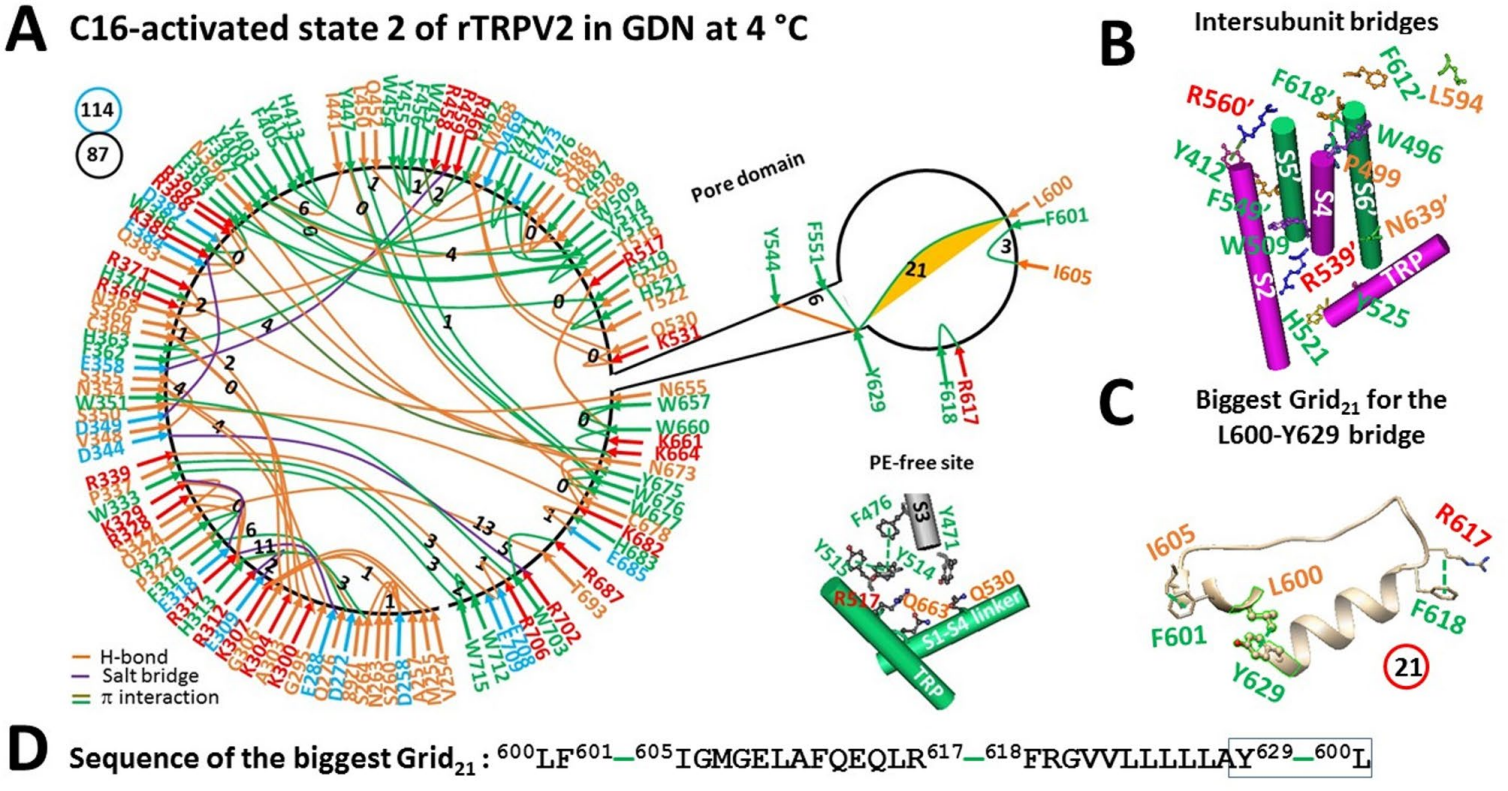
Thermoring structures of C16-activated state 2 of PE-free rTRPV2 at low temperature. (**A**) The grid-like noncovalently interacting mesh network along the PE-dependent minimal gating pathway of a single subunit of C16-activated state 2 of rTRPV2 in GDN at pH 8 and 4°C (PDB: 7ZJE). The same colored residues and arrows are used for convenient tracking. Salt bridges, π interactions, and H-bonds between paired amino acid side chains along the PE-dependent minimal gating pathway from V254 to P726 are denoted in purple, green, and orange lines, respectively. The specific constrained grid sizes necessary to regulate the least-stable noncovalent interactions in the grids are indicated with black numbers. The identified weakest L600-Y629 π bridge in the biggest Grid_21_ is emphasized in orange. The total grid sizes and the total grid size-controlled noncovalent interactions along the PE-dependent minimal gating pathway are displayed in cyan and black circles, respectively. The inset shows the structure of the PE-free site in the VSLD and at the vanilloid site. (**B**) Swapping interactions at the VSLD/PD’/PD interfaces near upper and lower gates along with the PE release in the C16-activated state 2. (**C**) The structure of the biggest Grid_21_ with a 21-residue size to regulate the weakest L600-Y629 bridge in the pore domain. (**D**) The sequence of the biggest Grid_21_ to control the weakest L600-Y629 bridge highlighted in the blue box.

In the second C16-activated state, the biggest Grid_21_ was born in the pore domain to govern the weakest L600-Y629 CH-π bridge (Fig. 6A), alongside the same swapping bridges found in the first C16-activated state (Figs. 5B and 6B). The corresponding thermoring Grid_21_ started from L600 to F601, I605, R617, F618, Y629, and back to L600 (Fig. 6C-D). For the weakest L600-Y629 bridge to be energetically equivalent to a basic H-bond (1.0 kcal/mol), the T_m, th_ was calculated as 32 °C (Table 2). Meanwhile, with changes in the total tertiary noncovalent bridges and grid sizes from 107 and 118 to 87 and 114, respectively, the mean structural thermosensitivity (Ω_10_) was calculated as 2.35 (Table 2), still lower than the initial heat sensitivity (Q_10_) of 132^12^. Consequently, the chemical perturbation at the vanilloid site but away from the putative heat sensors was insufficient to trigger cold activation at low temperature with a matched thermosensitivity to mirror the initial heat activation.

### Internal chemical perturbation away from the heat sensors induces stronger cold activation

**Fig. 7:**
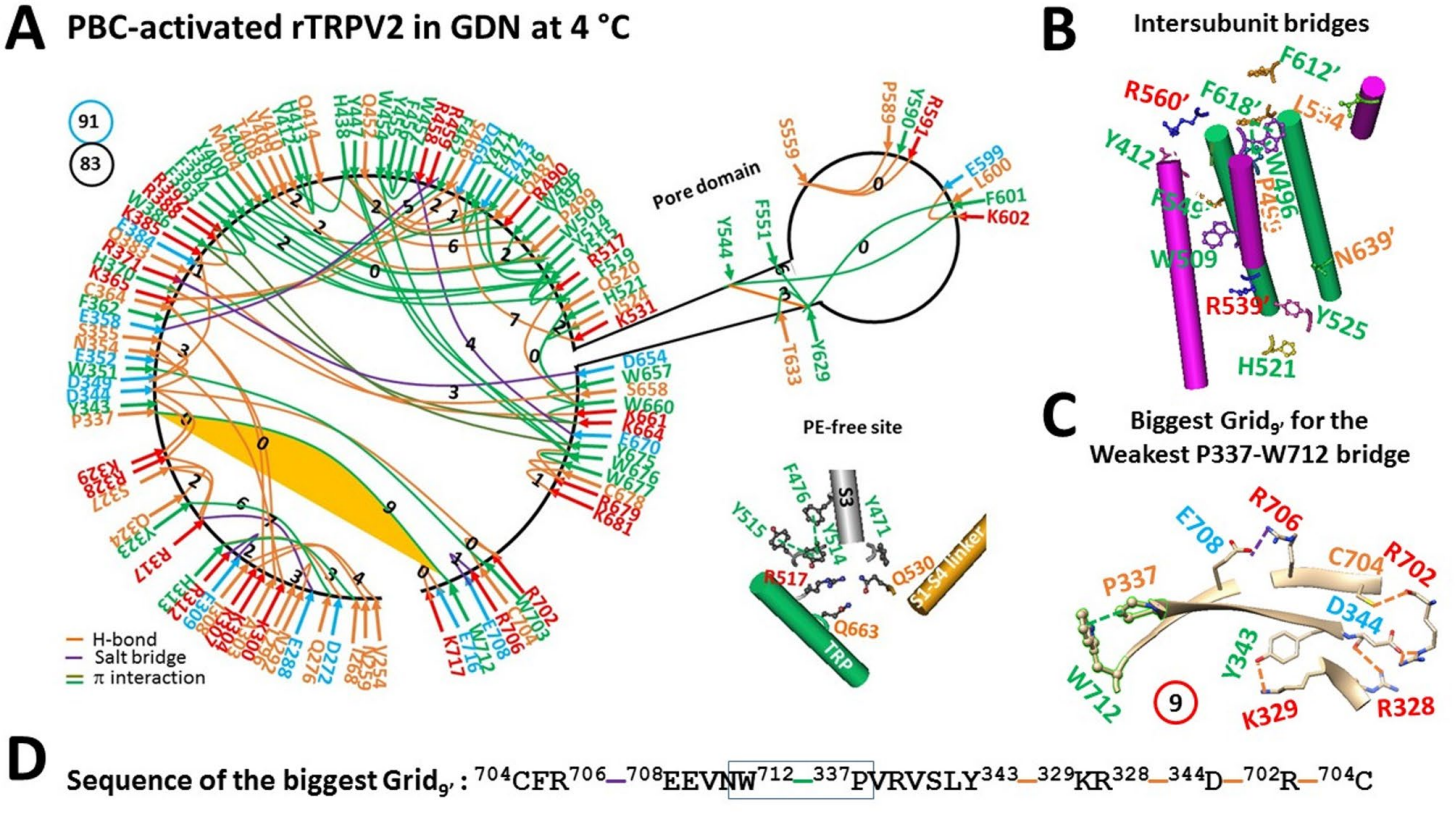
Thermoring structures of PBC-activated rTRPV2 at low temperature. (**A**) The grid-like noncovalently interacting mesh network along the PE-dependent minimal gating pathway of a single subunit of PBC-activated PE-free rTRPV2 in GDN at pH 8 and 4°C (PDB: 7ZJG). The same colored residues and arrows are used for tracking convenience. Salt bridges, π interactions, and H-bonds between paired amino acid side chains along the PE-dependent minimal gating pathway from V254 to P726 are denoted in purple, green, and orange lines, respectively. The specific constrained grid sizes necessary to regulate the least-stable noncovalent interactions in the grids are indicated with black numbers. The identified weakest P337-W712 π bridge in the biggest Grid_9’_ is emphasized in orange. The total grid sizes and the total grid size-controlled noncovalent interactions along the PE-dependent minimal gating pathway are displayed in cyan and black circles, respectively. The inset shows the structure of the PE-free site in the VSLD and at the vanilloid site. (**B**) Swapping interactions at the VSLD/PD’/PD interfaces near upper and lower gates along with the PE release in the PBC-activated state. (**C**) The structure of the biggest Grid_9’_ with a 9-residue size to regulate the weakest P337-W712 bridge at the interface between C- and N-terminals. (**D**) The sequence of the biggest Grid_9’_ to control the weakest P337-W712 bridge highlighted in the blue box.

In contrast, in response to the PBC perturbation at K118, L126, Y162, H165, I170, K174 from one subunit and W333’ and Y335’ from another neighboring subunit of rTRPV2^48^, only the swapping W496-F618’-P499 bridges remained intact. Following a significant decrease in the total tertiary noncovalent bridges and grid sizes from 107 and 118 to 83 and 91, respectively (Fig. 7A-B; Table S6), the systematic thermal instability (T_i_) was still 1.10 (Table 2), and the mean structural thermostability (Ω_10_) was calculated as 28.6 (Table 2). Among these tertiary noncovalent interactions, the P337-W712 π bridge near the PBC pocket became the weakest and was controlled by the biggest Grid_9’_ via a thermoring from C704 to R706, E708, W712, P337, Y343, K329, R328, D344, R702, C704 (Fig. 7C-D). Since this weakest bridge was energetically equivalent to a basic H-bond (1.0 kcal/mol), the calculated T_m, th_ was about 46 °C (Table 2). In that case, the PBC-induced cold activation below the threshold for heat sensing could not mirror the initial heat activation with matched thermosensitivity.

### Exposing the superficial heat sensors to a mild detergent causes maximal cold activation to mirror the initial heat activation

**Fig. 8:**
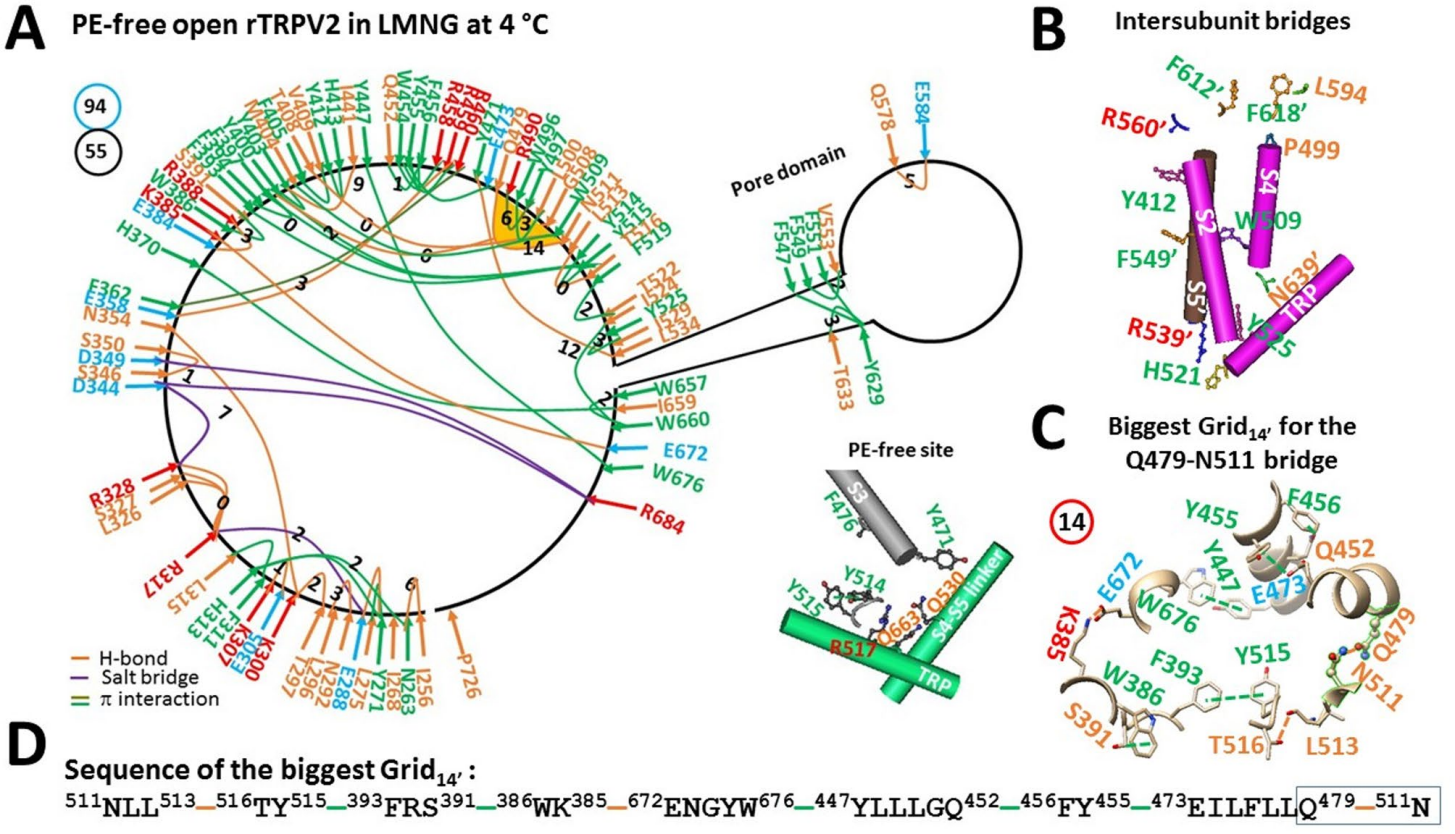
Thermoring structures of PE-free open rTRPV2 at low temperature. (**A**) The grid-like noncovalently interacting mesh network along the PE-dependent minimal gating pathway of a single subunit of open rTRPV2 in LMNG at pH 8 and 4°C (PDB: 6BO4). The same colored residues and arrows are used for convenient tracking. Salt bridges, π interactions, and H-bonds between paired amino acid side chains along the PE-dependent minimal gating pathway from V254 to P726 are marked in purple, green, and orange lines, respectively. The specific constrained grid sizes required to control the least-stable noncovalent interactions in the grids are labeled with black numbers. The identified weakest Q479-N511 bridge in the biggest Grid_14’_ is highlighted. The total grid sizes and the total grid size-controlled noncovalent interactions along the PE-dependent minimal gating pathway are shown in cyan and black circles, respectively. The inset shows the structure of the PE-free site in the VSLD and at the vanilloid site. (**B**) The swapping bridges at the VSLD/PD’/PD interfaces near the upper and lower gates are broken along with the PE release in the open state. (**C**) The structure of the biggest Grid_14’_ with a 14-residue size to control the weakest Q479-N511 bridge in the VSLD. (**D**) The sequence of the biggest Grid_14’_ to control the weakest Q479-N511 bridge highlighted in the blue box.

Given that the three heat sensors were located on the protein surface, it is reasonable that their exposure to a mild detergent LMNG opens the channel at 4 °C^43^. When compared with the putative pre-open closed state, not only was the primary weakest L555-Y590 π bridge in the pore domain disrupted, but also the second weakest S486-Y497 H-bond in the VSLD (Fig. 8A; Table S7). Although the third weakest K300-N354 H-bond was not broken (Fig. 8A), it was strengthened by the additional R328-D344 salt bridge so the T_m, th_ for both bridges was calculated as about 68 °C. Notably, most of the charge-involved noncovalent bridges such as D536-R539, E561-R617, R563-Q615, Y514-R517-W660, R490-H438, and D469-S466/R369 at the membrane surface were disconnected. Therefore, in addition to the detergent solubilization, the charged residues at the external and internal membrane-water interfaces were also hydrolyzed at low temperature. In this regard, all the swapping bridges in the putative pre-open closed state were disrupted for channel opening at both upper and lower gates (Figs. 4D and 8B). Further, the biggest Grid_14_ was found at the pre-S1/TRP/VSLD/pre-S1 interfaces to regulate the least-stable Q479-N511 H-bond. It had a 14-residue size via a thermoring from N511 to L513, T516, Y515, F393, S391, W386, K385, E672, W676, Y447, Q452, F456, Y455, E473, Q479, and back to N511 (Fig. 8C-D). Since the least-stable H-bond was energetically equivalent to 1.5 basic H-bond (1.5 kcal/mol), the calculated T_m, th_ was about 41 °C (Table 1), lower than the observed threshold of 48–55 °C for initial heat activation^3,11–13,15–18,20^. Therefore, this open state, once induced by a superficial exposure of at least three heat sensors to LMNG and hydrolysis of nearby charged residues at the external and internal membrane surfaces at pH 8 and 4 °C, could function as a cold-evoked open state below 41 °C.

Along the PE-dependent minimal gating pathway from V254 to P726, the total number of tertiary noncovalent interactions during channel opening decreased from 107 to 55. When these interactions formed a grid-like mesh network, the total number of grid sizes was 94, resulting in a systematic thermal instability (T_i_) of 1.71, which was higher than that of the putative stable pre-open closed state (Figs. 4A and 8A, Tables S3 & S7, Tables 1 and 2). Thus, this open state was relatively unstable. For the putative gating transition from the identified initial pre-open closed state to this open state, the calculated mean structural thermosensitivity (Ω_10_) was about 126, similar to the experimental functional thermosensitivity (Q_10_) of 132 (Table 2)^12^. Accordingly, this PE-free open state, along with a dramatic decrease in the total number of tertiary noncovalent bridges, could be used for unique cold activation to mirror the primary heat activation. In other words, the same heat capacity mechanism could be applied to illuminate the sharp heat response.

## Discussion

Rodent TRPV2 exhibits complex heat responses^3,11–13,15–18,20^. Despite its high initial temperature sensitivity (Q_10_ >100), the initial high activation threshold (> 50 °C) poses a challenge in capturing the heat-evoked open state using a cryo-EM approach at or above 50 °C. Therefore, the traditional temperature titration is insufficient to uncover the structural motifs underlying these complex heat responses^21,22^. On the other hand, the cryo-EM structures of TRPV2 in the closed and activated or open states at 4 °C are available in response to a variety of chemical perturbations^18,43,44,48–52^.

Given that the thermoring basis for the heat sensing of TRPV1, and TRPV3 and TRPV4 has been confirmed through their cryo-EM structures at various temperatures, and that both cold and heat activations from the same starting point exhibit mirrored thermosensitivity (Q_10_) when the heat capacity mechanism is applied^21–32^, thermoring analyses of these chemically-induced gating states were conducted in this computational study to define the specific thermal sensors and relevant intersubunit interctions for the initial heat sensitization of rTRPV2.

Since the calculated melting temperature thresholds (T_m, th_) of at least three weakest intrasubunit interactions on the protein surface in the putative stable pre-open closed state aligned well with the experimental thresholds (T_th_) for the initial heat activation, they could serve as the primary heat sensors. Further, cold activation induced by any chemical perturbation away from the putative heat sensors could not mirror the initial heat activation until simutaneous exposure of the superficial heat sensors to the mild detergent LMNG promoted hydolysis of nearby charged residues at the membrane surfaces for full channel opening.

Thereafter, the heat capacity mechanism, together with a change in relevant swapping interactions near the heat sensors, could be used to reveal the thermoring basis for the full rTRPV2 opening at both lower and upper gates along with the high heat sensitivity.

### Role of the first two PE-bound closed states

Along with two PE lipids in the VSLD and at the active vanilloid site in the first two closed states, the calculated melting thresholds were 33 °C and 46 °C (Table 1). Given the absence of the DSC transition before 47.7 °C^20^, and the calculated mean Ω_10_ was about -1.55 upon the release of PE from the vanilloid site, these two closed states may be coupled together. However, when the segment 358–369 of rTRPV2 is replaced with the counterpart 397–409 of rTRPV1 in the rTRPV2/V1(357–434) chimeric channel, the PE binding at the vanilloid site in these two closed states may be destabilized for channel activation separately at 34.5 °C and 46 °C, respectively, along with the small and major DSC transitions possibly upon the unfolding of the weakest E561-R617 and H438-R490 bridges^12,20^. Similarly, inserting a serine residue at position 365 of rTRPV2 may also destabilize the PE binding at the vanilloid site for channel activation at 46 °C^16^, along with the possible unfolding of the weakest H438-R490 π interaction in the second closed state (Fig. 3A-C). Finally, phosphorylation of Y335, Y471 and Y525 or oxidation of M528 and M607 may also destabilize the PE binding at the vanilloid site in these two closed states in favor of the initial activation at a lower threshold^15,17^. Alternatively, all the above genetic or chemical perturbations may also regulate the thermoring structures of the third closed state for channel activation at lower thresholds.

### Multiple heat sensors with matched thresholds for the initial heat activation and DSC transition

Recent thermoring studies have shown that the specific weakest noncovalent interaction is sufficient to account for the precise threshold for the heat activation of TRPV1, TRPV3 or TRPV4. For example, the heat-induced unfolding of the least-stable Y401-R499 or E406-K504 bridge at the pre-S1/VSLD interface is necessary to release the PI lipid from the active vanilloid site for rTRPV1 or hTRPV1 activation and the DSC transition above 43 °C^21,26,28^. Similarly, the heat-induced unfolding of the weakest R416-D519 salt bridge at the pre-S1/VSLD interface or the least-stable E467-K545 salt bridge at the interface between S1-S2 and S3-S4 linkers is responsible for releasing PC from the active vanilloid site to activate oxidized mTRPV3 or hTRPV3 with the C612-C619 disulfide bond above 40 °C or 30 °C^22,27,29,31^. However, in the reduced state, the heat-induced unfolding of the weakest K614-N647 or E610-K649 H-bond in the pore domain initiates the heat activation above 52 °C to activate mTRPV3 or hTRPV3 without the C612-C619 disulfide bond^23,24,29,30^. In the case of TRPV4, the weakest P498/Y502-Y567 bridge at the interface between S1-S2 and S3-S4 linkers has been identified for releasing an inhibitor from the VSLD for the heat activation above 24–35 °C^30^.

However, in this study, the release of PE from the active vanilloid site did not activate native rTRPV2. Instead, it created at least three least-stable tertiary noncovalent interactions on the protein surface, serving as primary heat sensors with a matched T_m, th_ range of 48 °C to 55 °C for the initial heat activation. These interactions included the S486-Y497 H-bond at the external interface between S1-S2 and S3-S4 linkers, the L555-Y590 π interaction at the external interface between two pore turrets and the K300-N354 H-bond at the internal pre-S1/ARD interface (Figs. 1, 4A-C and 10; Table 1)^3,11–13,15–18,20^. Therefore, the weakest K300-N354 bridge was unique among thermosensitive TRPV1-4 channels, leading to an extended gating pathway that covered the first ARD and the CTD (Figs. 2A, 3A, 4A, 5A, 6A, 7A and 8A; Tables S1-S7).

### Disrupting the swapping H521-R539’-Y525-N639’ and L594-F612’ bridges near the lower and upper gates is required for primary channel activation

Recent studies have shown that only one thermal sensor is allosterically linked with the highly conserved swapping π interaction between an aromatic residue on the S4-S5 linker and a positively charged residue on S5 near the lower gate for channel activation of TRPV1, TRPV3 or TRPV4. For example, the weakest Y401-R499/E406-K504 bridge and the swapping Y565-R579’ bridge in rTRPV1/hTRPV1, the weakest E610-K649 bridge and the swapping Y575-K589’ bridge in reduced hTRPV3, and the weakest P498/Y502-Y567 bridge and the swapping Y602-R616’ bridge in hTRPV4^30,31,53^.

However, in this study, disrupting the swapping H521-R539’ π bridge is insufficient to fully open rTRPV2. First, even if the H521A mutation disrupts the H521-R539’ π bridge, the initial heat activation is not affected^50^. Second, given that oxidation of M528 or M607 or phosphorylation of Y525 sensitizes the initial heat activation of rTRPV2^15,17^, the relevant swapping H521-R539’-Y525-N639’ and L594-F612’ bridges near the lower and upper gates in the putative pre-open closed state were broken even in the C16 or PBC-activated state (Figs. 5A, 6A and 7A). However, the lower and upper gates were not open simultaneously^18^. Therefore, disrupting not only the swapping L594-F612’/H521-R539’-Y525-N639’ bridges but also other intersubunit interactions is necessary for the full channel opening.

### Disrupting the swapping P499-F618’-W496 bridges at the VSLD/PD’ interface primes full TRPV2 opening

**Fig. 9:**
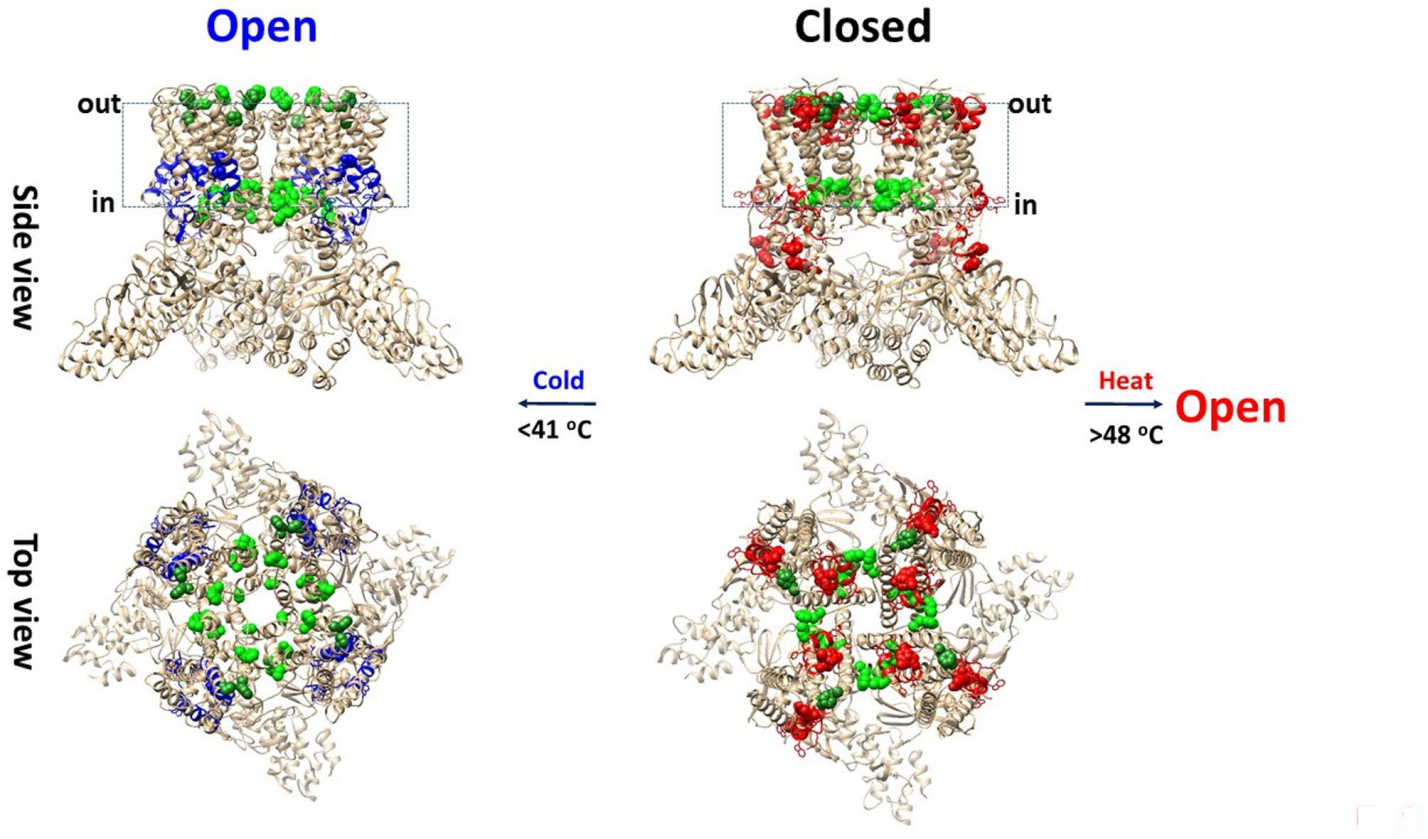
Working model of the thermodynamic coupling between cold and heat activations of rTRPV2. The homo-tetrameric cryo-EM structures of rTRPV2 in the PE-bound closed state 3, and the PE-free open state at 4°C (PDB ID, 8EKR and 6BO4, respectively) are used for the model. The weakest intrasubunit S486-Y497 (top red), L555-Y590 (top, red) and K300-N354 (bottom, red) bridges, as well as the intersubunit L594-F612’ (top, green), F518’-P499 (top, dark green) and H521-R539’-Y525-N639’ (middle, green) are shown in the pre-open closed state for initial heat above 48 °C or cold activation below 41 °C. When these bridges are broken but the K300-N354 bridges are enhanced upon simultaneous exposure to a mild detergent LMNG and hydrolysis of charged residues at the membrane surface below 41 °C, the channel is fully open along with the weakest Q479-N511 bridges (blue) near the lower gate, as well as high thermosensitivity (Q_10_>100) to mirror the initial heat activation.

Given the frequent disruption of the swapping bridges H521-R539’-Y525-N639’ and L594-F612’ at or near heat-sensitive oxidation or phosphorylation sites at Y471, Y525, M528 or M607 during cold activations with varing thermosensitivities (Figs. 2D, 3D, 4D, 5B, 6B, 7B and 8B; Table 2), it is evident that other intersubunit interactions need to be disconnected for the channel to fully open with high thermosensitivity. Further research demonstrated that the swapping P499-F618’ and/or F618’-W496 interactions were also situated close to the weakest S486-Y497 and L555-Y590 bridges in the primary closed states 1–3. These interactions persisted in the C16- or PBC-activated state but were disrupted in the C16 and PBC-activated state or the LMNG-opened state (Fig. 9)^18^. In contrast, the corresponding R455-E600’ swapping bridges were present in the cold-evoked open state (PDB: 8U3L) but were broken in the closed and open states at 48 °C (PDB: 7LPC and 7LPE, respectively)^21,54^. Therefore, not only the swapping bridges H521-R539’-Y525-N639’ and L594-F612’ near the lower and upper gates but also the swapping P499-F618’-W496 interactions at the VSLD/PD’ interfaces must be disrupted for the channel to fully open with the matched high thermosensitivity.

### Cold activation with matched thresholds and thermosensitivity supports the heat capacity model

Recent studies have shown that removing PI from the active vanilloid site disrupts the weakest Y401-R499 and H410-I696 bridges at the VSLD/pre-S1/TRP interfaces. This leads to the broken swapping of the Y565-R579’ bridges near the lower gate for both cold and heat activations of reduced rTRPV1-Δ(604–826) with a shared thermosensitivity around 21^30,54,55^. Similarly, releasing PC from the active vanilloid site also disconnects the weakest E467-K545 bridges at the S1-S2/S3-S4 linker interface, resulting in the broken swapping of the Y575-K589’ bridges near the lower gate for both cold and heat activations of oxidized mTRPV3 with a mirrored thermosensitivity around 3^14,27,31^. In contrast, the K169A mutation, located far away from the weakest E610-K649 salt bridge in the pore domain, induces a slow cold activation of reduced TRPV3 that is significantly different from the fast heat activation^23,30,56^. Taken together, these results are consistent with the heat capacity model for symmetric cold and heat activations from the same starter^32^.

In this study, it was found that releasing PE from the active vanilloid site is unnecessary for rTRPV2 activation^44^. However, the calculated Ω_10_ of 126 for the putative gating transition from the PE-bound closed state 3 to the PE-free cold-evoked open state upon exposure of three superficial heat sensors to LMNG and hydrolysis of nearby charged residues at the membrane surface was close to the experimental Q_10_ of 132 for the initial heat activation^12,43^(Figs. 4 and 8; Table 2). In contrast, the calculated Ω_10_ values from 2.35 to 14.6 or 28.6 for the putative gating transition from the PE-bound closed state 3 to the C16 or PBC-activated state were much lower than 132 when the C16 or PBC site was away from three putative heat sensors (Figs. 4, 5, 6 and 7; Table 2)^12,18^. Thus, the heat capacity mechanism may be responsible for the very high thermosensitivity of the primary heat activation.

### Model limitations and the scope of its applicability

The proposed heat capacity model for the high heat sensitivity of rTRPV2 is based on matched heat thresholds in the third resting closed state with PE bound and mirrored cold sensitivity between this PE-bound closed state and the detergent-solubilized, charged residues-hydrolyzed and PE-free open state at 4 °C. However, it is unclear if this closed state in the MSP2N2 nanodiscs remains at body temperature (37 °C) and can be opened above 48 °C or below 41 °C directly by disrupting or enhancing the same identified weakest noncovalent bridges at the interfaces between the pre-S1 domain and the ARD, between the S1-S2 and S3-S4 linkers and between the two pore turrets. In addition, the lower resolution of the open state (PDB, 6BO4) also limits the accurate application of the thermoring model. Further structural and functional data with higher resolution or molecular dynamic simulations including tertiary π interactions above 48 °C or below 41 °C along with mutations are necessary to further examine these models.

## Conclusions

The intricate protein-lipid interactions in thermosensitive TRP channels present challenges in capturing the specific cryo-EM structures that dictate their roles in heat or cold sensing. In this computational study, the tertiary structural changes of TRPV2 from resting to activated or open states were quantified by using a high-sensitivity thermoring model. By aligning predicted thresholds, comparing thermosensitivity between cold and heat activations, locating intersubunit interactions near the thermosensors, and correlating thermostability with published experimental data, one could identify at least three thermosensors on the TRPV2 surface to start mirrored heat and cold activations. Therefore, this model, in line with the heat capacity mechanism, undoubtedly serves as a powerful tool in understanding thermo-gated TRPV2 and similar receptors.

## Supplementary Information

Below is the link to the electronic supplementary material.

Supplementary Material 1

## Data Availability

All data generated or analysed during this study are included in this published article and supplementary material.

## Abbreviations

ARD: Ankyrin repeat domain
cryo-EM: Cryogenic electron microscopy
CTD: C-terminal domain
DMNG: Decyl maltose neopentyl glycol
DSC: Differential scanning calorimetry
GDN: Glyco-diosgenin
LMNG: Lauryl maltose neopentyl glycol
MSP2N2: Membrane scaffold protein 2N2
PE: Phosphatidylethanolamine
PC: Phosphatidylcholine
PI: Phosphatidylinositol
C16: Phytocannabinoid Δ9-tetrahydrocannabiorcol
PD: Pore domain
PH: Pore helix
PBC: Probenecid
Ω_10_: Structural thermosensitivity
Q_10_: Functional thermosensitivity
T_i_: Systematic thermal instability
T_m, th_: Melting temperature threshold
T_th_: Activation threshold
TRP: Transient receptor potential
TRPV: TRP vanilloid
TRPVi: (i = 1, 2, 3, 4)
hTRPV1: Human TRPV1
hTRPV4: Human TRPV4
hTRPV3: Human TRPV3
mTRPV3: Mouse TRPV3
rTRPV1: Rat TRPV1
rTRPV2: Rat TRPV2
VSLD: Voltage-sensor-like domain
