# Supplementary material for: Thermodynamic coupling between cold and heat activations of TRPV2
Source: Sci Rep. 2026 Jun 10;16:18024. doi: 10.1038/s41598-026-54781-w (PMC13254303; doi:10.1038/s41598-026-54781-w)
Supplement: Supplementary file 1 — Supplementary Material 1 [file 41598_2026_54781_MOESM1_ESM.pdf]

Supporting Information for

Thermodynamic coupling between cold and heat activations of TRPV2

Guangyu Wang 1, 2*

[1]Department of Physiology and Membrane Biology, University of California School of Medicine, Davis, CA, USA

[2]Department of Drug Research and Development, Institute of Biophysical Medico-chemistry, Reno, NV, USA

* Correspondence: gary.wang10@gmail.com

This supplementary material includes:

Tables S1, S2, S3, S4, S5, S6 and S7.

Table S1. Tertiary noncovalent interactions along the PE-dependent minimal gating pathway from V254 to P726 in each subunit of PE-bound closed state 1 of rTRPV2 in DMNG/MSP2N2 at pH 8 and 4 °C (PDB ID, 8EKP).

Noncovalent interaction	Cut-off distance	Linked residues
Salt bridge	3.2-4 Å	E288-R317, D344-R702, E358-R459, K531-D654, R535-E536-R539, R535-E647, E614-R619
H-bond	<3.9 Å donor-H-acceptor <60°	V254/A257-K307, I256-N263, D258-S260/N263, Y271-E288, D272-H313, N292-L296, K300-N354, A303/K304-C364, G306-N368, E309-R312, Q316-E376, R317-L326/R328, E318-T331, Q324-S327, P325/S327-R702, R328-S346, E332-R706, D344-S346, S347-V356, D349/S350-K385, D349-E685, S350-E672, S366-N368, R369-D469, H370-S658, Q383-D387, R388-E685, N396-Y675, Y400-Q479/N511, T408-W509, Y447-W677, R458-E670, S466-S470, L475-N511, Q479-S504, Q487-R490, Y471-PE-R517-PE-Q530-PE-Q663-PE-R517, R517-W660, M528-K531, R535-R539, E561-R617, E599-K602, L600-Y629, D654-S656, N655-S658, K690-G692, R702-D700
π−π interaction	2.65–6.5 Å	F362-F462, W386-F393-Y515, F394-F519, F397-Y515, Y400-Y403-Y407, Y400-F476, Y400-Y515, Y447-W676, W454-W676, Y455-F456/F462, Y471-F472-Y514, F476-Y514/Y515, Y514-Y515, F540-Y544, Y544-F601, F551-Y629-F603, W676-W677, W712-W715
cation−π interaction	<6.0 Å	W351-R684-W703, W386-K664, H438-R490, W454-R458, W457-R460
CH_3/CH−π interaction	2.65-3.01 Å	I268-H313, Q276-Y323, F330-V691, P337-W712, H370-I659, L381-F705, L450-W676, F476-N511, R490-Y497, Q520-W660, Y525-I529, F540-M640, F547-L636, F549-V553, L555-Y590, F603-V630, W657-K661, W660-K664, L688-W703, W715-L719
Lone pair−π interaction	3-3.7 Å	Y455-E473, Y629-T633

Table S2. Tertiary noncovalent interactions along the PE-dependent minimal gating pathway from V254 to P726 in each subunit of PE-bound closed state 2 of rTRPV2 in DMNG/MSP2N2 at pH 8 and 4 °C (PDB ID, 8EKQ).

Noncovalent interaction	Cut-off distance	Linked residues
Salt bridge	3.2-4 Å	E309-R312, D344-R702, D536-R539
H-bond	<3.9 Å donor-H-acceptor <60°	V254/M255/A257-K307, D258-S260/N263, Y271-E288-R317, D272-H313, N292-L296, K300-N354, A303/K304-C364, G306-N368, Q316-E376, R317-L326/R328/K329, E318-T331, Q324-S327/V691, P325/S327-R702, R328-S346, E332-R706, D344-S346, S347-V356, D349/S350-K385, D349-E685, S350-E672, E358-R459, S366-N368-R371, R369-D469, E376-K380-R706, Q383-D387, K385-E672/E685, R388-E685, N396-Y675, Y400-G508, Y403-I441, T408-W509, Q414-H438, Y447-W677, Q452-E473, R458-E670, S466-D469/S470, T516-T522, Y471-PE-R517-PE-Q530-PE-Q663-PE-R517, R517-W660, R535-E536, Y544-Y629, Y590-A611, S592-D595, E599-A611, L600/T604-Y629, N673-K682, R702-D700
π–π interaction	2.65–6.5 Å	F362-F462, W386-F393-Y515, F394-F519, F397-Y515, Y400-Y403, Y400-F476, Y400-Y515, Y447-Y675/W676, W454-W676, Y455-F456, Y471-F472-Y514, F476-Y514/Y515, Y514-Y515, Y544-F601, F551-Y629, W676-W677, W712-W715
cation–π interaction	<6.0 Å	R684-W703, W386-K664, R392-Y675, H438-R490, W454-R458, W457-R460
CH_3/CH–π interaction	2.65-3.01 Å	I268-H313, Q276-Y323, F330-V691, P337-W712, W351-R684, L381-F705, L450-W676, R490-Y497, Q520-W660, V543-F547, F547-L632, F549-V553, L555-Y590, F603-L610, Y629-T633, V630-Y634, W657-K661, W660-K664, L688-W703
Lone pair–π interaction	3-3.7 Å	Y455-E473

Table S3. Tertiary noncovalent interactions along the PE-dependent minimal gating pathway from V254 to P726 in each subunit of PE-bound closed state 3 of rTRPV2 in DMNG/MSP2N2 at pH 8 and 4 °C (PDB ID, 8EKR).

**Noncovalent interaction**	**Cut-off distance**	**Linked residues**
Salt bridge	3.2-4 Å	E309-R312, D344-R702-D700, E358-R459, R369-D469, R388-E685, D536-R539
H-bond	<3.9 Å donor-H-acceptor <60°	A257-K307, N259-N368, **Y271-E288-R317**, D272-H313, R280-Y323, **N292-L296**, **K300-N354**, A303/K304-C364, Q316-E376, **R317-L326/R328**/K329, F319-S327-Q324, P325/S327-R702, R328-S346-S347/D344, E332-R687, C334-G336, S350-E672, H370-S658, V374-N379, N396-Y675, Y447-W677, R458-E670, S466-D469, Q479-T483, S486-Y497, Y514-R517-W660, T516-T522, Y544-Y629, E561-R617, R563-Q615, S592-D595, E599-K602, E672-R680, T693-T698, R694-P699, R706-E708, N711-A713, T721-S723-D725
π–π interaction	2.65–6.5 Å	F362-F462, W386-**F393-Y515**, F394-F519, F397-Y515, **Y400-Y403**, Y400-F476, **Y400-Y515**, Y403-F407, F405-W509, **Y412-H413**, **Y447-**Y675/**W677**, **Y455-F456**, Y471-F472-Y514, F476-Y514/Y515, **Y514-Y515**, F540-Y544, Y544-F601, **F551-Y629**, F603-Y629, W657-W660, W676-W677, W712-W715
cation–π interaction	<6.0 Å	W351-R684-W703, W386-K664, H438-R490, W454-R458, W457-R460
CH_3/CH–π interaction	2.65-3.01 Å	I268-H313,Q276-Y323, **F311-L315**, F330-V691, W333-P726, P337-W712, **M404-W509**, V410-H438, L450-W676, **Y455-R459**, F467-Y471, Q520-W660, V543-F547, **F549-V553**, L555-Y590, L600-Y629, W657-K661, W660-K664, L688-W703
Lone pair–π interaction	3-3.7 Å	F601-T604, Y629-T633

Note: Bold interactions were conserved in closed state 3 and the open state.

Table S4. Tertiary noncovalent interactions along the PE-dependent minimal gating pathway from V254 to P726 in each subunit of PE-free activated state 1 of rTRPV2 with C16 bound in GDN at pH 8 and 4 °C (PDB ID, 7ZJD).

**Noncovalent interaction**	**Cut-off distance**	**Linked residues**
Salt bridge	3.2-4 Å	E309-R312, D344-R702, D349-K385, R369-D469, E384-K388, K385-E672, R459-E670, R535-D536/E647
H-bond	<3.9 Å donor-H-acceptor <60°	V254/M255/A257-K307, S260-E262, Y271-E288-R317, D272-H313, N292-L296, K300-N354, A303/K304-C364, R317-L326/R328/K329, E318-T331, P325/S327-R702, S327-K329, E332-R706, S341/Y343-E709, V348-S355, E352-S355, C364-S366/R369, N368-R371, N379-K661, E384-R687, N396-Y675, Y400-N511/Q479, T408-W509, Y447-W677, R458-E670, Q479-N511, S486-Q487, Y514-R517-W660, D536-R539, Y544-Y629, S559-P589, S592-L594, M671-G674, N673-R680
π–π interaction	2.65–6.5 Å	F362-F462, W386-F393-Y515, F393-F394, Y400-Y403, Y400-F476, Y400-Y515,Y412-H413, Y447-W676, W454-W457/W676, Y455-F456, Y471-F472-Y514, F472-F476-Y514/Y515, Y514-Y515, F519-H521, Y544-F601, F551-Y629, Y590-F612, W657-W660, W676-W677, W712-W715
cation–π interaction	<6.0 Å	H438-R490, W454-R458, R539-F540
CH_3/CH–π interaction	2.65-3.01 Å	I268-H313, Q276-Y323, W333-V340, P337-W712/W715, W386-K664, F395-C399, F397-Y515, M404-W509, V410-H438, L450-W676, R490-Y497, F540-M640, F547-L632, L555-F612, F603-L610, R617-F618, W657-K661, W660-K664, K690-W703
Lone pair–π interaction	3-3.7 Å	Q276-F319, W351-E685, F476-N511

Table S5. Tertiary noncovalent interactions along the PE-dependent minimal gating pathway from V254 to P726 in each subunit of PE-free inactivated state 1 of rTRPV2 with C16 bound in GDN at pH 8 and 4 °C (PDB ID, 7ZJE).

Noncovalent interaction	Cut-off distance	Linked residues
Salt bridge	3.2-4 Å	E288-R317, E309-R312, E318-K329, D344-R702, D349-K385, E358-R459
H-bond	<3.9 Å donor-H-acceptor <60°	V254/M255/A257-K307, D258- S260/N263/S264, D272-H313, G295/K300-N354, A303/K304-C364, G306-N368, R317-S327/R328/K329, Q324-T693, S327-K329, R339-E709, V348-S355, S350-K682, C364-S366, N368-R371, R369-D469, Q383-D387, E384-K388, R392-Y675, N396-Y675, Y400-G508, Y403-I441, Y447-W677, Q452-E473, M468-Q530/K531, S486/Q487-Y497, T516-T522, R517-W660, Q530-K531, Y544-Y629, N673-K682, C678-Y675, H683-E685, R706-E708
π−π interaction	2.65−6.5 Å	W333-W715, W351-W703, F362-H363-F462, W386-F393-Y515, F394-F519, F397-Y515, Y400-F476, Y400-Y515, F405-W509, Y412-H413, Y447-W676, W454-W676, Y455-F456, Y471-F472-Y514, F476-Y514, Y514-Y515, F551-Y629, W657-W660, W676-W677, W712-W715
cation−π interaction	<6.0 Å	W454-R458, W457-R460
CH_3/CH−π interaction	2.65-3.01 Å	I268-H313, P322-Y323, P337-W712, W386-K664, L450-W676, Q520-H521, L600-Y629, F601-I605, R617-F618, W657-K661, W660-K664, R687-W703
Lone pair−π interaction	3-3.7 Å	Q276-F319

Table S6. Tertiary noncovalent interactions along the PE-dependent minimal gating pathway from V254 to P726 in each subunit of PE-free activated state of rTRPV2 with PBC bound in GDN at pH 8 and 4 °C (PDB ID, 7ZJG).

Noncovalent interaction	Cut-off distance	Linked residues
Salt bridge	3.2-4 Å	E288-R317, E309-R312, E358-R459, R371-D654, R458-E670, R706-E708
H-bond	<3.9 Å donor-H-acceptor <60°	V254-K307, N259-I308, D272-H313, N292-L296, K300-N354, A303/K304-C364, R317-S327/R328, Q324-S327, R328-D344, K329-Y343, D344-E352, D349-S355/K385, D344-R702-C704, K365-S466/D469, H370-S658, Q383-K661, E384-K388, T408-W509, V409-H413, Q414-Y497, H438-Q487, Q452-E473, D469-K531, R517-W660, Y544-Y629, S559-P589/Y590/R591, E599-K602, W676-R679, W677-R679, C678-K681, E716-K717
π–π interaction	2.65–6.5 Å	W351-W703, F362-F462, W386-F393-Y515, F393-F394-F519, F397-Y515, Y400-F476, Y400-Y515, F405-W509, Y412-H413, Y447-Y675/W676, W454-W457/W676, Y455-F456, Y471-F472-Y514, F476-Y514/Y515, Y514-Y515, Y544-F601, F551-Y629, W657-W660, W676-W677
cation–π interaction	<6.0 Å	W386-K664, R392-Y675, H438-R490
CH_3/CH–π interaction	2.65-3.01 Å	I268-H313, Q276-Y323, P337-W712, M404-W509, V410-H438, W496-P499, Y514-R517, H521-I524, L600-Y629, W660-K664
Lone pair–π interaction	3-3.7 Å	Y629-T633

Table S7. Tertiary noncovalent interactions along the PE-dependent minimal gating pathway from V254 to P726 in each subunit of PE-free open rTRPV2 in LMNG at pH 8 and 4 °C (PDB ID, 6BO4).

Noncovalent interaction	Cut-off distance	Linked residues
Salt bridge	3.2-4 Å	**E288-R317**, R328-D344-R684-D349
H-bond	<3.9 Å donor-H-acceptor <60°	I256-M263, **Y271-E288**, **N292-L296**, T297-K300, **K300-N354**, E305-K307, **R317-L326/S327/R328**, S346-S350, E358-R459, E384-R388, K385-E672, Y400-G508, Y403-I441, T408-W509, Y471-L534, Q479-N511, L513-T516, Q578-E584
π–π interaction	2.65–6.5 Å	Y271-H313, **F393-Y515**, F394-F397-Y515, **Y400-Y403**, **Y400-Y515**, **Y412-H413**, **Y447-W676**, **Y455-F456**, **Y514-Y515**, F547-Y629, **W657-W600**
cation–π interaction	<6.0 Å	F362-R460, R490-Y497
CH_3/CH–π interaction	2.65-3.01 Å	Y271-L275, **F311-L315**, H313-I268, H370-I659, W386-S391, **M404-W509**, F405-V409, W454-R458, **Y455-R459**, W496-L500, I524-W660, Y525-I529, **F549-V553**, **Y629-F551,** Y629-T633
Lone pair–π interaction	3-3.7 Å	Y455-E473, F519-T522

Note: Bold interactions were conserved in closed state 3 and the open state.
